# The Usefulness of DEPAP Flaps for Reconstructing Perineal Defects Caused by Fournier’s Gangrene: A Case Report

**DOI:** 10.3390/jcm14248732

**Published:** 2025-12-10

**Authors:** Dong Gyu Kim, Kyung Ah Lee

**Affiliations:** Department of Plastic and Reconstructive Surgery, Inje University Haeundae Paik Hospital, Busan 48108, Republic of Korea; donkey0317@gmail.com

**Keywords:** Fournier’s gangrene, deep external pudendal artery perforator flap, perineal defect

## Abstract

**Background**: Fournier’s gangrene is a rare and aggressive form of necrotizing fasciitis involving the perineal, genital, and perianal regions. Despite advances in critical care, early diagnosis and rapid surgical intervention remain crucial to reduce mortality and morbidity. Extensive debridement often leads to complex perineal defects that require reliable reconstructive options. This study presents a case highlighting the usefulness of the deep external pudendal artery perforator (DEPAP) flap in perineal reconstruction following Fournier’s gangrene. **Methods**: A 43-year-old male patient developed Fournier’s gangrene secondary to underlying colon cancer scheduled for chemotherapy. Following wide excision and serial debridement to remove necrotic tissue, reconstruction was performed using a DEPAP flap designed from the upper medial thigh region. The flap was elevated based on perforators identified by a handheld Doppler and rotated to cover the perineal defect. **Results**: The flap survived completely without any vascular compromise or wound complications. The patient achieved satisfactory functional recovery with stable wound healing and an acceptable cosmetic outcome. No recurrence or contracture was observed during the 9-month follow-up. **Conclusions**: Fournier’s gangrene associated with underlying colon cancer and subsequent chemotherapy presents additional challenges due to impaired wound healing and increased infection risk. In such complex cases, the deep external pudendal artery perforator (DEPAP) flap offers a reliable single-stage reconstructive option that ensures durable coverage, rapid recovery, and minimal donorsite morbidity. Our case demonstrates that even in immunocompromised or oncologic patients, the DEPAP flap provides stable wound healing and satisfactory functional and esthetic outcomes, supporting its usefulness in managing perineal defects after oncologic Fournier’s gangrene.

## 1. Introduction

Fournier’s gangrene is a rare yet highly aggressive disease, characterized by necrotizing fasciitis affecting the external genitalia, perineum, or perianal area. It accounts for only a small fraction of surgical emergencies, representing approximately 0.02% of annual hospital admissions and an estimated incidence of around 1.6 cases per 100,000 males per year, with rates exceeding 3 per 100,000 in men aged 50–79 years. Contemporary series still report case-fatality rates ranging from roughly 5% to 40%, underscoring its clinical importance despite advances in critical care [1]. This rapidly spreading condition requires urgent medical attention and timely interventions to prevent life-threatening complications [2].

Fournier’s gangrene typically arises from local infections in the genital or perianal region and can rapidly progress to severe tissue necrosis, systemic sepsis, and even multiple organ failure.

Patients with predisposing factors, such as diabetes mellitus, immunosuppression, chronic alcoholism, obesity, and peripheral vascular disease, are at a higher risk of developing Fournier’s gangrene. Additionally, individuals with underlying colorectal malignancies may be more susceptible to this condition. Malignancy-related Fournier’s gangrene, particularly when caused by perforated or locally advanced rectal and rectosigmoid cancers, is increasingly recognized as a distinct subset with more complex therapeutic decisions and worse outcomes than benign etiologies. In such patients, timely recognition of the underlying tumor and coordinated oncologic management are crucial to improving long-term prognosis. Large cohort studies have shown that these comorbidities not only predispose to the disease but also worsen prognosis; for example, diabetes mellitus and other immunosuppressive states roughly double to triple the odds of adverse outcomes, late presentation beyond 48 h from symptom onset increases the odds of death by approximately three- to fourfold, and systemic inflammatory response at admission is associated with an even higher odds of mortality [3,4].

The treatment for Fournier’s gangrene involves aggressive resuscitation, administration of broad-spectrum antibiotics, and surgical debridement. Early and extensive surgical debridement is essential to control infection and prevent further spread. Multiple debridement procedures may be necessary to ensure complete removal of infected tissue [5].

Following debridement, patients develop large perineal defects that require reconstruction. Some reconstructive procedures are used in Fournier’s gangrene to provide skin coverage with functional and cosmetic results and less morbidity. The choice of reconstruction method depends on several factors, such as the location and size of the defect and patient’s medical condition [6].

Herein, we present a case of Fournier’s gangrene caused by underlying colon cancer in a patient scheduled for chemotherapy.

## 2. Case Description

**Figure 1:**
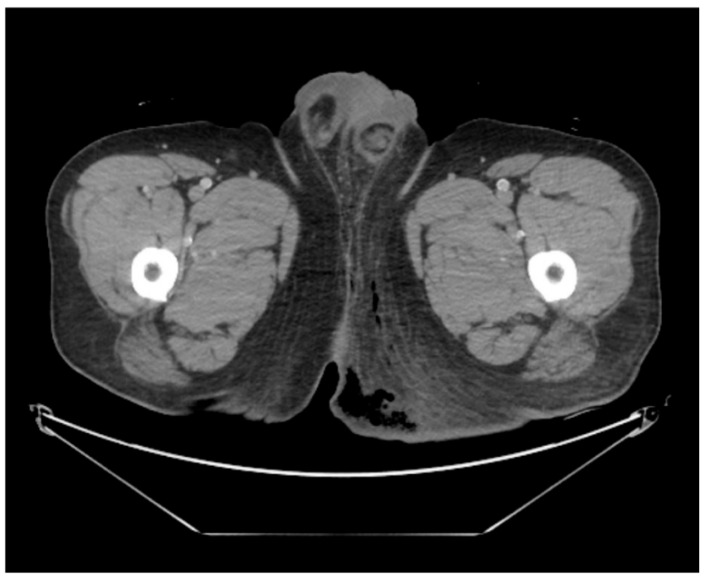
Pelvic computed tomography scan in emergency room. The image reveals subcutaneous emphysema with fat infiltration in the left buttock and scrotum, suggesting Fournier’s gangrene.

This case was conducted in accordance with the principles of the Declaration of Helsinki and was approved by the Institutional Review Board of Inje University Haeundae Paik Hospital (IRB No. 2023-12-028-001). Written informed consent for publication of clinical details and images was obtained from the patient. A 43-year-old male patient presented to the emergency department complaining of severe perineal pain and pus discharge from multiple sites in the perineum. The patient had undergone surgery for fistula at another clinic a week ago. He reported a medical history of diabetes, hypertension, and heavy alcohol consumption. Physical examination revealed an edematous scrotum with a foul-smelling exudate. Pelvic computed tomography (CT) revealed air collection in the buttock area, indicating Fournier’s gangrene (Figure 1). Baseline laboratory tests demonstrated marked leukocytosis (white blood cell count 22.74 × 10^9^/L) with neutrophilia, elevated C-reactive protein (30.34 mg/L), and an increased serum lactate level (2.5 mmol/L), consistent with sepsis. Glycated hemoglobin was 7.2% and fasting glucose was 208 mg/dL, reflecting suboptimal glycemic control in a patient with known diabetes. Due to the aggressive nature of the condition and the development of sepsis, broad-spectrum antibiotics were promptly initiated. Subsequently, the patient underwent radical debridement in both the General Surgery and Urology departments.

**Figure 2:**
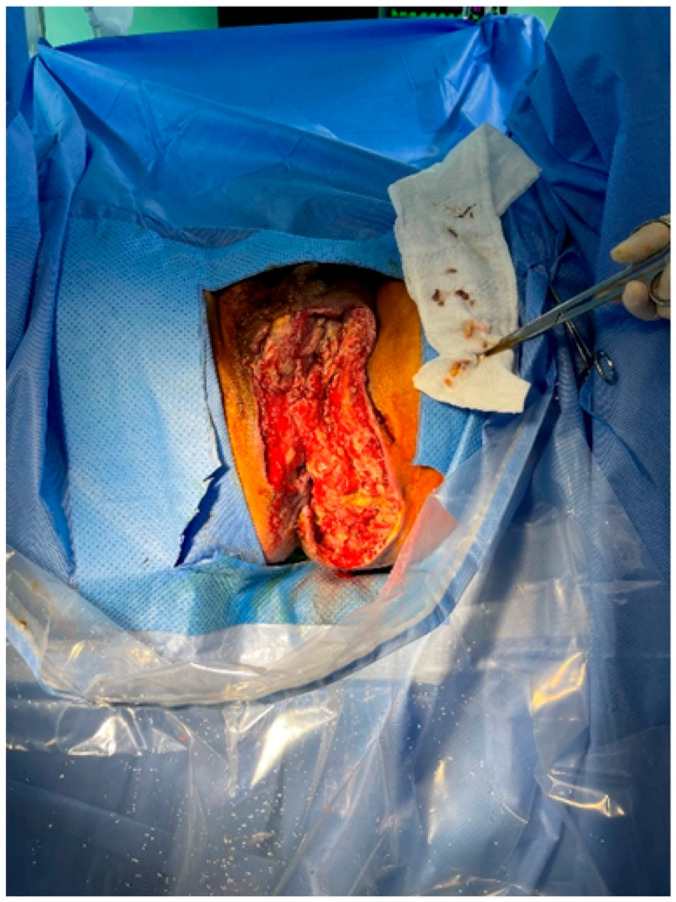
Intraoperative photo taken upon the patient’s referral to the Plastic Reconstruction Department. Extensive perineal defects and residual necrotic tissue were observed after debridement. Subsequently, serial debridements were performed.

**Figure 3:**
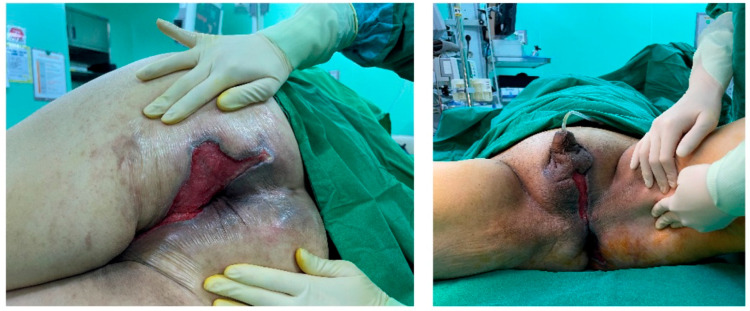
Clinical image after 4 weeks of negative pressure wound therapy (NPWT). Debridement and NPWT were performed twice a week for 4 weeks, after which colostomy and reconstruction were scheduled.

Following serial debridement at the General Surgery Department, the patient was referred to the Plastic and Reconstructive Surgery Department for infection control and reconstruction of the extensive perineal defect (Figure 2). Negative pressure wound therapy (NPWT) was used to aid in wound healing and reduce defect size. During the course of NPWT for 4 weeks, a decrease in wound size and formation of healthy granulation tissue without any signs of infection was observed (Figure 3). During follow-up, abdominal computed tomography (CT) revealed a suspicious region consistent with colon cancer. Further pathological examinations confirmed the diagnosis of rectosigmoid colon cancer. Histopathology demonstrated a moderately differentiated adenocarcinoma of the rectosigmoid colon, staged as cT3N1M0 on cross-sectional imaging. The General Surgery (GS) team strategized a treatment plan involving colostomy and stoma surgery to address the underlying colon cancer. Following surgery, the patient was scheduled to undergo chemotherapy as part of a comprehensive treatment approach, with a planned FOLFOX-based protocol after recovery from sepsis and wound reconstruction. This integrated strategy aimed to manage both Fournier’s gangrene and the underlying cancer, maximizing the chances of a successful outcome for the patient.

**Figure 4:**
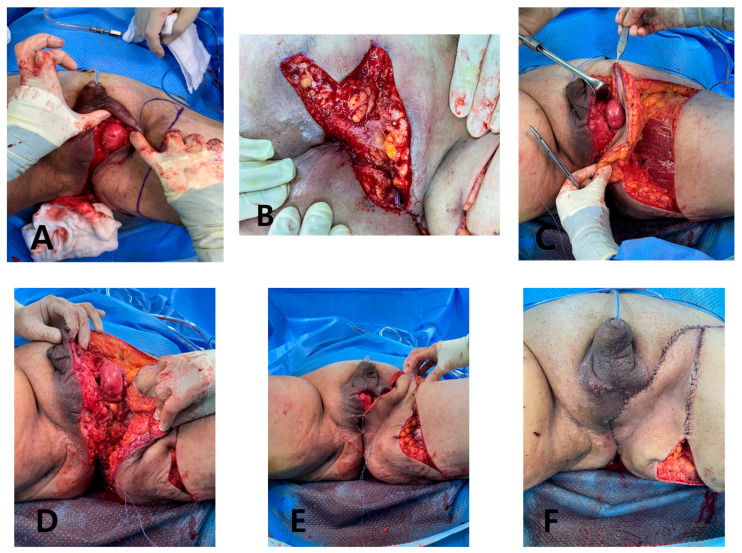
Intraoperative and postoperative views of DEPAP flap reconstruction. (**A**) Anterior view of the extensive scrotal defect with exposure of the perineal structures after serial debridement. (**B**) Posterior view demonstrating the defect extending to the perianal region. (**C**) Anterior view showing elevation and insetting of the deep external pudendal artery perforator (DEPAP) flap toward the perineal defect. (**D**) Anterior view after flap insetting, demonstrating resurfacing of the scrotal and perineal region. (**E**) Intraoperative assessment of the reconstructed scrotal area after insetting, confirming adequate flap position and contour. (**F**) Appearance during the reconstruction, showing stable coverage of the defect and restoration of the perineal and scrotal contour.

Under general anesthesia, the GS team performed further debridement and colostomy. Because of the extensive defect measuring 25 × 11 cm from the scrotum to the posterior anal area, we opted for a deep external pudendal artery perforator (DEPAP) flap for reconstruction. The DEPAP flap, based on the posterior pudendal thigh flap, offers well-vascularized tissue ideal for wound healing and minimizes the risk of complications. With the patient in the lithotomy position and the hip slightly flexed and abducted, the flap was designed over the upper medial thigh along the groin crease. The main perforator was explored and identified in the septum of the groin crease using a handheld Doppler. Flap elevation began at the distal end of the subfascial plane, and careful dissection was performed to preserve the integrity of the perforator and the surrounding vascular structures. The flap was dissected from the medial thigh to the posterior scrotal level, achieving an adequate arc of rotation toward the perineal defect. Once the DEPAP flap was adequately dissected, it was transposed to cover the extensive perineal defect resulting from the previous surgeries. The DEPAP flap is a fasciocutaneous flap that enables direct closure of the donor site. The flap was positioned and secured to the surrounding healthy tissue with meticulous suturing to ensure proper wound closure and minimize tension on the flap (Figure 4).

Functional, esthetic, and patient-reported outcomes were prospectively evaluated during scheduled outpatient visits up to 9 months postoperatively. At each visit, the surgeons inspected the flap for stability, hypertrophic scarring, or contracture and documented perineal discomfort, ability to sit and ambulate, and continence in the medical record. Patient-reported satisfaction was assessed qualitatively using open-ended questions regarding pain, ease of perineal hygiene, return to daily activities, and overall satisfaction with the reconstructive result.

## 3. Results

The patient was placed in the supine position with the thigh adducted to relieve tension for 7 days to minimize movement. Drains were removed without any observed side effects such as hematoma or infection; therefore, we encouraged the patient to start walking gradually after 7 days. The skin sutures were removed on postoperative day 14, at which time the flap was fully viable with no evidence of congestion, partial necrosis, or wound dehiscence. Since no adverse effect was noted, the patient was discharged from the hospital 20 days after surgery.

**Figure 5:**
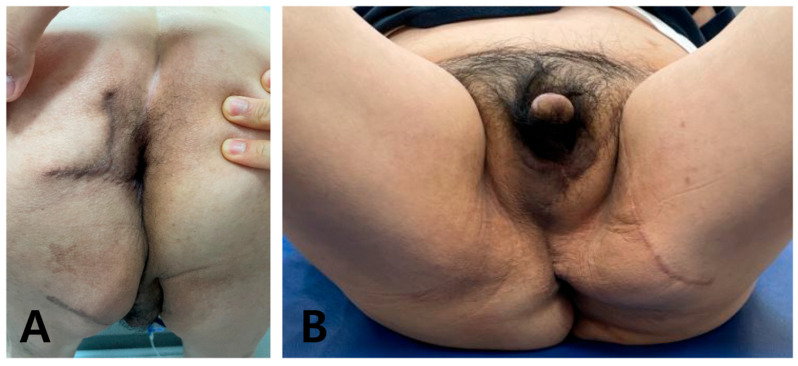
The patient showed satisfactory progress during the postoperative period for 9 months. The flap remained completely viable without any functional impairment, and the patient’s rectal mucosal and anal sphincter functions were preserved without complications (**A**). An anterior view demonstrates the absence of tension on the reconstructed area and excellent restoration of the scrotal contour (**B**).

Over a 9-month follow-up period, the patient reported no difficulties with sitting, walking, or performing daily perineal hygiene, and he denied perineal pain at rest or during ambulation. Anal sphincter tone and rectal mucosal integrity remained intact on clinical examination, with no evidence of flap contracture or deformity. The DEPAP flap remained fully viable without congestion, partial necrosis, or wound dehiscence throughout follow-up, and no donor-site complications were observed. Overall, the patient expressed a high level of satisfaction with both the functional recovery and the cosmetic appearance of the reconstructed perineal region (Figure 5).

## 4. Discussion

Fournier’s gangrene is an infrequent yet life-threatening emergency characterized by swiftly spreading necrotizing infections affecting the perineum and external genitalia. This condition is typically polymicrobial and involves a mixture of aerobic and anaerobic organisms, as illustrated in detailed case descriptions by Kordahi and Suliman [7] and in larger clinical series [1,2,8], and it poses a substantial risk of septic shock and multi-organ failure if not promptly and effectively managed.

Early diagnosis is pivotal for improving patient outcomes. The clinical symptoms of Fournier’s gangrene include intense pain, erythema, edema, and crepitus in the affected areas. Patients may also manifest systemic signs of infection, such as fever, tachycardia, and leukocytosis [9]. A heightened level of suspicion is crucial, especially in individuals with predisposing factors, such as diabetes mellitus, immunosuppression, and underlying colorectal malignancies, as they are at an elevated risk of developing Fournier’s gangrene [10].

Malignancy-related Fournier’s gangrene represents a distinct and particularly challenging subset. Several reports have shown that perforated or locally advanced rectal and rectosigmoid cancers can serve as the primary source of perineal sepsis, and these patients tend to present later, with more extensive soft-tissue involvement and higher rates of septic shock than those with benign etiologies [10,11]. In their review of rectal cancer-associated Fournier’s gangrene, Bruketa et al. emphasized that oncologic patients frequently have multiple predisposing factors, including diabetes, malnutrition, and immunosuppression from chemotherapy, which compound the risk of poor wound healing and mortality [10]. In such cases, prompt recognition of the underlying colorectal malignancy, timely diversion or resection, and coordinated planning of subsequent oncologic treatment are crucial to improving long-term outcomes [10,11].

The initial management of Fournier’s gangrene involves aggressive resuscitation with broad-spectrum antibiotics administration. Surgical intervention is the cornerstone of the treatment for Fournier’s gangrene. Emergency debridement of necrotic tissue is essential to control the infection and prevent further spread. Therefore, serial debridement may be necessary in such cases. However, these procedures often lead to defects in the perineal area [8]. Reconstruction of scrotal and perineal defects after surgical debridement is typically achieved using skin grafts or flaps; however, as summarized in the systematic review by Karian et al. [12] and subsequent series [6,8], no consensus exists on a single optimal method of defect reconstruction. The choice of reconstructive procedure should hinge on the location and size of the defect, the presence of exposed vital structures, the degree of contamination, the patient’s medical condition, and the surgeon’s experience [6]. Small, superficial scrotal defects with preserved tunica vaginalis can often be managed with split-thickness skin grafts or local advancement flaps, whereas large composite defects extending to the perineum or anal region usually require well-vascularized flap coverage to protect exposed structures, fill dead space, and tolerate ongoing contamination or planned adjuvant therapy [6,12]. In medically fragile or oncologic patients who are likely to receive chemotherapy, reconstructive strategies must balance robust tissue coverage with a limited operative time and acceptable donor-site morbidity. In practice, surgeons also tend to favor techniques with which they have consistent experience and predictable pedicle anatomy, as this reduces intraoperative ischemia time and technical complications in an already unstable population.

From a review of the literature, several authors have reported reconstruction of scrotal and perineal defects after Fournier’s gangrene using split-thickness skin grafts, local scrotal or medial thigh advancement flaps, pudendal thigh flaps, gracilis muscle flaps, and vertical rectus abdominis or other musculocutaneous flaps [6,8,11,12]. In the systematic review by Karian et al., which included 16 studies with 425 patients, 22.6% of defects were reconstructed with skin grafts, 16.0% with scrotal advancement flaps, and 30.1% with regional or distant flaps, with the remaining patients managed by secondary intention, delayed primary closure, or combined techniques; overall, most approaches provided acceptable functional and cosmetic results but no single method emerged as clearly superior [12]. Skin grafts are technically simple and widely available, but they may contract and provide insufficient bulk or protection for exposed perineal structures, especially in patients who require prolonged sitting or adjuvant chemotherapy [6,12]. Musculocutaneous flaps offer robust, well-vascularized tissue, yet they are associated with greater donor-site morbidity, longer operative times, and, in some cases, limited reach to the posterior anal region, which can be problematic in septic or medically fragile patients [6,8,11,12]. Perforator-based pudendal and perineal flaps have therefore gained popularity, as they preserve underlying muscle function, reduce donor-site morbidity, and provide favorable color and thickness match while allowing primary closure of the donor site in many cases [13].

Compared with previously reported cases, our patient was relatively young but had multiple risk factors, including poorly controlled diabetes and rectosigmoid cancer requiring colostomy and planned FOLFOX-based chemotherapy. For a 25 × 11 cm defect extending from the scrotum to the posterior anal area, techniques such as split-thickness skin grafting or musculocutaneous flaps were considered less suitable because of the risks of graft failure, contracture, and higher donor-site morbidity in the setting of severe sepsis and anticipated chemotherapy. We selected a deep external pudendal artery perforator (DEPAP) flap because it provides thick, well-vascularized fasciocutaneous tissue with color and texture similar to the perineal skin, has a reliable pedicle with an adequate arc of rotation to reach the posterior anal region, and allows primary closure of the donor site [13,14]. In this case, these characteristics translated into uneventful wound healing, preservation of perineal and sphincter function, and a high level of patient satisfaction over nine months of follow-up, while completing reconstruction without delaying the planned oncologic treatment.

Taken together, the existing literature highlights the variety of reconstructive options after Fournier’s gangrene but offers limited data on flap selection in malignancy-related cases that require early oncologic treatment. Our report adds to the current evidence by demonstrating that a DEPAP flap can be safely used to reconstruct a large perineal defect after malignancy-related Fournier’s gangrene in a patient with multiple comorbidities. This case suggests that the DEPAP flap is a useful option when reliable, low-morbidity soft-tissue coverage is needed in a contaminated perineal field to facilitate timely progression to adjuvant chemotherapy.

The DEPAP flap offers several benefits over alternative reconstruction techniques. It provides well-vascularized tissue with a color similar to that of the perineal area, diminishing the risk of flap failure. Additionally, the DEPAP flap has potential sensory characteristics and shares the same nerve supply, enhancing functional outcomes. Notably, the operative time for DEPAP flaps is shorter than that for free flaps, thus minimizing surgical complexity [14]. Perforator-based pudendal and perineal flaps have been increasingly adopted for reconstruction of oncologic and infectious perineal defects because they combine reliable vascular anatomy with limited donor-site morbidity and favorable color and thickness match [13]. Internal and external pudendal artery perforator flaps can be harvested in the supine position, rotated to reach the scrotum, perineum, and posterior anal region, and closed primarily at the donor site in many patients, which shortens operative time and facilitates postoperative care [13]. The deep external pudendal artery perforator flap, as described in phalloplasty and perineal reconstruction series [14], provides sensate, well-vascularized fasciocutaneous tissue that can be tailored to defect size while preserving underlying muscle function. In our case, these characteristics were particularly advantageous in a high-risk oncologic patient who required stable coverage over a contaminated perineal field and timely initiation of adjuvant chemotherapy.

Overall, the DEPAP flap proved to be a reliable and effective option for reconstructing extensive perineal defects in this challenging case. Its success underscores the importance of considering individual patient factors and selecting an appropriate reconstruction method to achieve optimal outcomes.

## 5. Conclusions

Fournier’s gangrene is rare in patients with underlying colon cancer scheduled for chemotherapy. The comprehensive treatment approach involving colostomy and subsequent chemotherapy aims to manage both Fournier’s gangrene and the underlying cancer, thus enhancing the chances of a successful outcome for the patient. However, this treatment process limits the perineal reconstruction method for defects.

A DEPAP flap offers a reliable solution, providing well-vascularized tissue to promote wound healing and minimize complications. This flap, based on the posterior pudendal thigh flap, demonstrated its efficacy in the present case, leading to successful reconstruction without functional impairment.

## Data Availability

The original contributions presented in this study are included in the article. Further inquiries can be directed to the corresponding author.

## Abbreviations

DEPAP	the deep external pudendal artery perforator
NPWT	negative pressure wound therapy
